# Gustatory Alteration Evaluation in Patients with Chronic Otitis Media

**DOI:** 10.1016/S1808-8694(15)30495-X

**Published:** 2015-10-19

**Authors:** Felippe Felix, Shiro Tomita, Basìlio de Bragança Pereira, Jamerson Reis Cordeiro, Guilherme Carleti, Fernando de Souza Barros, Gustavo Augusto Porto Sereno Cabral

**Affiliations:** 1 Graduate Student (MSc) - Federal University of Rio de Janeiro - Hospital Universitàrio Clementino Fraga Filho da UFRJ; 2 Full Professor of Otorhinolaryngology - UFRJ, Head of the ENT Department - HUCFF - UFRJ; 3 Full Professor of Biostatistics - UFRJ, Coordinator of the Statistics Support Group - Hospital Universitàrio Clementino Fraga Filho - UFRJ; 4 Medical Studet - UFRJ - Scientific Initiation Discipline - Otorhinolaryngology - UFRJ; 5 Medical Studet - UFRJ - Scientific Initiation Discipline - Otorhinolaryngology - UFRJ; 6 Medical Studet - UFRJ - Scientific Initiation Discipline - Otorhinolaryngology - UFRJ; 7 Medical Studet - UFRJ - Scientific Initiation Discipline - Otorhinolaryngology - UFRJ

**Keywords:** cholesteatoma, suppurative otitis media, taste

## Abstract

Many studies have shown the consequent gustatory alteration caused by ear surgeries. However, few have reported this alteration in patients with chronic otitis media (COM), prior to surgical treatment.

**Aim:**

to identify gustatory alterations due to chorda tympani nerve involvement in patients with COM without prior surgery.

**Methods:**

Clinical essay, with tests based on “taste strips” with different concentrations of salt, sweet, bitter, and sour, was performed in 45 patients with unilateral cholesteatomatous or suppurated COM not previously submitted to otological surgery, using the disease-free ear on the contralateral side as control. The score ranged between 0 and 16.

**Results:**

A total of 25 patients presented cholesteatoma and 20 had non-cholesteatomatous disease. The mean score was 6.65 for the affected side and 9.93 for the half of the tongue on the side of the healthy ear (p<0.001). No patients had complained of gustatory alterations before the examination. Among the 24 patients with unilateral hypogeusia, eight cases of unilateral ageusia were found on the affected side. There was an association between cholesteatoma (p=0.055), disease duration (p=0.07) and worsening in gustatory sensitivity.

**Conclusion:**

Patients with COM can have gustatory alterations, even in the absence of complaints.

## INTRODUCTION

During ear surgery, the chorda tympani nerve is constantly exposed and it can be stretched or even cut during the procedure. These events may cause dysgeusia, hypogeusia or even ageusia, which are most of the times transitional complaints during postoperative.

Many studies have shown the consequent change in taste after ear surgery, aiming at treating chronic otitis media and otosclerosis^1^, ^2^, ^3^. Nonetheless, very few studies have reported taste alterations as a sign or symptom of patients with chronic otitis media prior to surgery.

This type of change was reported by Vlasto^4^, in 1930. Ho^5^ also described this finding in a small group of cases he reported. Arnold^6^ showed clinical evidence of the vulnerability of the chorda tympani nerve in patients with chronic disorders of the middle ear. After this period, very few studies reported such findings.

## OBJECTIVES

To assess the presence of gustatory alterations in the area innervated by the chorda tympani nerve in patients with chronic otitis media who have not yet been submitted to surgery.

## MATERIALS AND METHODS

### Study Place and Model

This is a clinical trial held at the Chronic Otitis Media Ward of the Otorhinolaryngology Department of this institution. The study was approved by the Ethics in Research Committee of the University Hospital, under protocol # 106/06.

### Patient Selection and Sample

 

### Eligibility Criteria

Individuals with unilateral chronic otitis media seen at this hospital from July of 2005 through September of 2007, who fit the following criteria:

### Inclusion criteria:

Patients from both genders, older than 18 years, regardless of race, who meet the inclusion criteria, with:

1. Unilateral cholesteatomatous middle ear chronic disease - clinically and radiologically confirmed, with a healthy contralateral ear waiting for surgery at this hospital.
2. Unilateral chronic non-cholesteatomatous otitis media with effusion (Lillie type II), clinically and radiologically confirmed with a healthy contralateral ear, waiting for surgery at this hospital.

### Exclusion Criteria:

1. Patients previously submitted to ear surgery in any of the sides.
2. Patients with prior history of facial paralysis
3. Patients with active tongue disease.
4. Patients with neurological disorders which impair cognition or communication.
5. Patients using some medication which can alter taste.
6. Patients without mastoid and middle ear CT scan.

The patient was initially selected by an otorhinolaryngologist, based on clinical signs and symptoms of chronic otitis media and with a CT scan proving the disorder. The diagnosis of chronic cholesteatomatous or non-cholesteatomatous otitis media was based on physical examination, CT scan and surgical findings.

Besides the test, the patient answered a questionnaire with personal information about his/her past of ear diseases, the duration of this middle ear chronic disease (considered as of the onset of symptoms), the presence of systemic diseases, smoking and use of medication.

### About the procedure

The test was carried out with a gustatory test validated by Muller et al.^7^ based on “taste strips” with different concentrations of salt, sweet, bitter and sour, and two strips with water, making up 18 strips. The concentrations were: sour - citric acid 0.3 g/ml, 0.165 g/ml, 0.09 g/ml, and 0.05 g/ml; bitter - quinine sulphate 0.006 g/ml, 0.0024 g/ml, 0.0009 g/ml and 0.0004 g/ml; sweet- sucrose 0.4 g/ml, 0.2 g/ml, 0.1 g/ml and 0.05 g/ml; salt- sodium chloride 0.25 g/ml, 0.1 g/ml, 0.04 g/ml and 0.016 g/ml. These strips were made out of filter paper, measuring 8cm in length and an area of 0.2 cm^2^. We established two different test orders: A and B, employed in a random fashion to the patients.

We chose patients with only one side affected by the chronic otitis media so that the other half of the tongue could be used as control in this study.

### The procedure

The patient can not eat food or drink liquids (except for water) for at least 1 (one) hour before the test or smoke on the day of the test. The examiner does not know which is the ear with chronic otitis media and also the taste of the filter paper which is being tested.

The filter paper strips are prepared with the taste in another room, being a total of 18 for each side. The strip is placed on half of the tongue which is being assessed and the patient must point to a chart to which of the five options (salt, sweet, bitter, sour, water) he/she believes to be correct, without bringing the tongue back to the oral cavity. The test is repeated in the same sequence on the opposite side.

In between each strip tasted, water is given to the patient so that we wipe out all residue of the last taste. At the end, a score from 0 to 16 is given to each side, and the two water strips are used to assess the test. Scores lower than 8 characterize hypogeusia and a score of 0 (zero) means ageusia.

### Data Processing and Analysis

The data collected was analyzed through the R software, and we used the multivariate analysis, using the Poisson Regression and the Wald test (z), was used to control confusion factors.

The models created through the Poisson regression were compared by means of the Akaike information criterion^8^. This criterion was proposed for selection, within a set of models, and the best is the one which has the lowest AIC value.

Moreover, we used a survival curve analogy by the Kaplan-Meyer technique, where instead of years of survival, we used taste tests correct answers. In order to test the survival curves we applied the Log-Rank test, the Tarone-Ware and the Peto and Prentice tests for each explanatory variable individually.

## RESULTS

We assessed 45 patients with mean age of 38 years varying from 19 to 70 years. 16 patients were men. 23 patients had the left side affected and the rest had otologic disease on the right side.

No patient reported any taste change prior to the questionnaire, nor xerostomia. Moreover, during the test no patient had any oral cavity or tongue lesion.

When we compared the healthy tongue side with the side with otologic disease in relation to gustatory sense, we found an average of 6.65 to the ipsilateral side and chronic otitis media and 9.91 to the side of the healthy ear (see Table 1).

**Table 1:** Results from the gustatory tests, average of correct answers and number of patients with hypogeusia or ageusia in each side.

	Normal side (mean+SD)	Sick side (mean+SD)	Hypogeusia Normal side	Hypogeusia Sick side	Cases of ageusia
NC COME	9.90+3.56	7.15+4.74	7	9	3
CCOM	10.2+3.61	6.28+4.82	5	15	5
Total	9.93+3.53	6.65+4.74	12	24	8

We found 24 cases of hypogeusia in the affected side and 8 (17.7%) cases of ageusia. On the healthy ear side we found 12 cases of hypogeusia and no case of ageusia.

**Figure 1:**
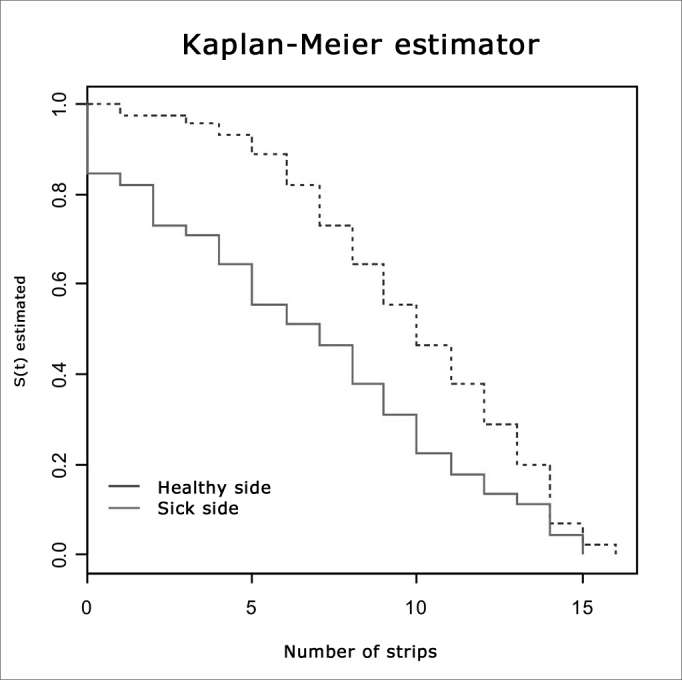
Survival Analysis Graph by the Kaplan-Meyer technique analyzing the results from the taste test for each side.

Through the Kaplan-Meyer survival graph we do not see crossover between the healthy and sick sides (see Fig. 1), confirmed by the tests presented on Table 2. The likelihood that the chronic middle ear disease is correct in answering × or more strips is always lower in the healthy side ear.

**Table 2:** Tests confirming Fig. 1 findings in the survival analysis:

Tests	P
Tarone-Ware test	p= 0.00239
Log-rank test	p= 0.00908
Peto&Prentice test	p= 0.000943

**Figure 2:**
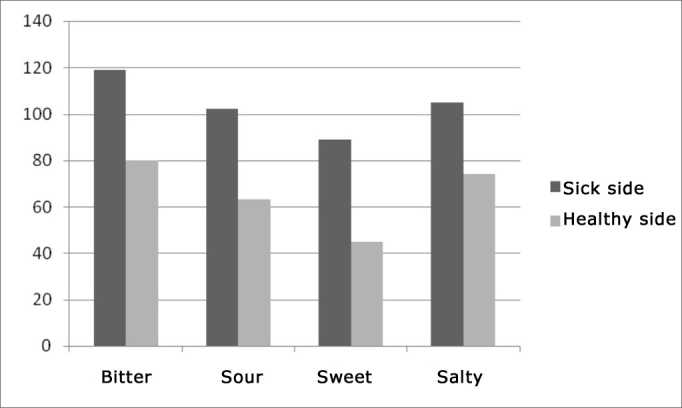
Comparing number of errors for each taste type between the two sides.

When we analyze each taste separately, the sweet one was more often felt in the damaged side when compared to the other tastes (see Table 3) and the one most affected was the bitter taste. The greatest confusion happened between bitter and sour. On the healthy side, we also have the bitter taste with the highest number of errors and the one most preserved was the sweet (see Fig. 2). The two most often mistaken tastes were also salty and sour, followed by sour and bitter.

**Table 3:** Number or errors for each taste offered (horizontal bar) on the sick side and the number of wrong answers (horizontal bar).

Sick side	Bitter	Sour	Sweet	Salty	Water	Total
Bitter	0	17	12	6	84	119
Sour	38	0	10	18	36	102
Sweat	10	8	0	4	67	89
Salt	8	35	10	0	52	105

We assessed the relationship of the same variables and their influence in altering the gustatory sensation through the analysis of different Poisson regression models (see Table 4). After analyzing other regression models, this one was chosen for having the lowest AIC and this, with its variables, will be adopted as significant, in other words, not a mere chance. The variables used were: gender, age, presence of otorrhea at the time of the test, smoking, diabetes mellitus, cholesteatoma and ear disease duration.

**Table 4:** Relationship between taste alteration and the different variables analyzed through the Poisson Regression.

	Estimate	Standard error	z Value	p Value	Code
Interseptum	3,169772	0,143455	22,096	<0,001	***
Sick side	-0,396536	0,074669	-5,311	<0,001	***
Age	-0,15032	0,002888	-5,205	<0,001	***
Male gender	-0,230843	0,083034	-2,780	0,00543	**
Smoking	-0,237583	0,104638	-2,271	0,02318	*
Disease duration	-0,046434	0,025907	-1,792	0,07307	
Cholesteatoma	-0,150241	0,078371	-1,917	0,05523	
Diabetes	0,307271	0,109366	2,810	0,00496	**

Sign. of the codes: 0 *** 0.001 ** 0.01 * 0.05. 0.1 1

AIC: 531.86

Males proved to be more sensitive to taste alterations than women (see Table 4). As with gender, age seems to negatively influence gustatory sensation: the older the patient, the greater the taste alteration.

Diabetes mellitus proved to be related to taste (see Table 4). We did not find any relationship between arterial hypertension and a worsening in gustatory sensation (p>0.1) in the Tarone-Ware test.

**Figure 3:**
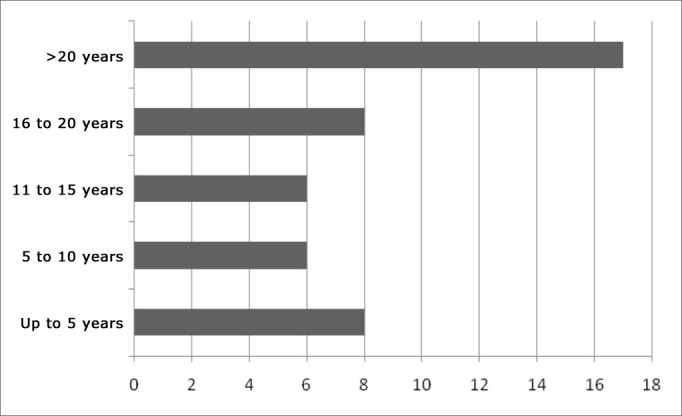
Number of patients according to ear disease duration.

Cholesteatoma present in 25 cases seemed to influence taste alteration in our sample (p=0.055). By the same token, disease duration (see Fig.3), patients with ear problems of longer duration seem to have a worsening in their tasting ability (p=0.073).

## DISCUSSION

One of the most important findings in our study was to notice that there is taste alteration on the half of the tongue of the same side of the chronic otitis media, when compared to the healthy side. Finding such taste alterations can justify the most frequent complaint of taste disorders after otosclerosis surgery, clearly shown by many surgeons, when compared to the post-op of patients with chronic otitis media, in other words, the latter group already had some previous alteration.

When we compare our results with those from a similar study, such as the one by Landis et al.^9^ as we break down the two types of COM, patients with cholesteatoma, more specifically, have an average score of 9.04 for the diseased side compared to 6.25 found in our study. Considering non-cholesteatomatous patients, the results for the same side of the disease was 7.15 in our study and 10 found by Landis et al.^9^. Sano et al.^11^ found similar results using electrogustometry. The scores found in our study were lower, maybe due to the very characteristic of our population, dependent on public care, who wait long times in lines for proper diagnosis and surgical treatment.

Complete ageusia was found in 8 cases in our study and Landis et al.^9^ found 9 patients with the same alteration. Despite the clear alteration in gustatory function on the same side of the COM, there was no complaint of taste disorder among these patients, even those who had ageusia. This feeling can be explained by the gustatory perception of the mouth in general and not only sectorial. Taste buds are located at different points of the oral cavity. Sensitivity is transported by different nerves and they all converge to the nucleus solitarius at the brainstem. Studies in rats11 show that gustatory afferences from the oral cavity and tongue have a mutual inhibition, in other words, the pathway which transmits most of the stimuli inhibits the others. This same study suggests that the loss of a great part of gustatory afferences (e.g. such as the case of a damage to the chorda tympani nerve) causes a central inhibition. Therefore, other areas with functioning gustatory afferences would receive a greater importance centrally, compensating the loss of the affected area, keeping the gustatory perception constant even when considerable areas are affected. Thus, even if a patient with COM does not complain of dysgeusia, this does not mean that there is no measurable gustatory failure.

Landis et al. in their two papers^9^, ^12^ already proposed to ask for a taste evaluation of patients among the preoperative evaluation tests prior to middle ear surgeries, for medical-legal reasons based on the findings of this study.

When we compare the gustatory test technique used in the different studies, Sano et al.^10^ used electrogustometry contrary to what we and Landis et al.^9^ did, we used natural tastes in filter paper strips. Although the electrical stimulation technique is simpler to perform, it produces predominantly metallic or acid sensations, and not the other types of tastes. Moreover, there is a current discussion about the possible co-stimulation through the electrical stimuli of trigeminal nerve fibers or about the direct activation of axons from the gustatory fibers doing a bypass of the tongue's taste buds^13^.

In our analysis, cholesteatomas seem to worsen gustatory sensation, just like Landis et al.^9^ found out. This data was expected, since cholesteatomas can cause bone destruction and facial paralysis. Thus, one would expect that the chorda tympani nerve would be affected in a greater number of patients with cholesteatomas. Now, Sano et al.^10^ did not find such relationship. Studies^14^, ^15^with temporal bones support this opinion arguing that cholesteatomas represent a greater damage to the chorda tympani nerve when compared to bones with chronic inflammatory non-cholesteatomatous processes. In order to emphasize the importance of the inflammatory process and its relationship with taste, Bartoshuk et al.^16^ reported that they noticed that children with repetition otitis media have a lower number of taste buds when compared to normal individuals.

In our study, disease duration seemed to have the same relation with the worsening in gustatory sensation. Sano et al.^10^, in their study, did not find relationship between disease duration - cholesteatomatous or not - with any worsening in gustatory sensation. Landis et al.^9^ did not analyze this issue in their study.

The taste more sensitive to the chorda tympani nerve in our study was the bitter taste. Sweet taste was the option with the lowest number of errors made in the test. Both, sweet and bitter presented the same type of intracellular activation through receptors coupled to the Protein G which activate intracellular second-messengers. One of the tastes which was most mistaken both in the healthy and impaired side was salty with sour, which act on the same way when they trigger taste buds through the direct activation of ionic channels on the cell surface. This finding was also noticed by Mueller et al.^7^ and Ahne et al.^17^. Nonetheless, the other case of confusion between tastes happened between bitter and sour, which have different activation pathways and there is no explanation for it so far.

## CONCLUSION

The data presented in this study show that patients with chronic middle ear inflammation have reduced gustatory sensation in their anterior two-thirds of the tongue ipsilateral to the disease, when compared to normal ears.
